# Differences in the early stages of motor learning between visual-motor illusion and action observation

**DOI:** 10.1038/s41598-023-47435-8

**Published:** 2023-11-16

**Authors:** Katsuya Sakai, Tsubasa Kawasaki, Yumi Ikeda, Junpei Tanabe, Akari Matsumoto, Kazu Amimoto

**Affiliations:** 1 Department of Physical Therapy, Faculty of Health Sciences, Tokyo Metropolitan University, 7-2-10, Higashiogu, Arakawa-ku, Tokyo, Japan; 2 Department of Physical Therapy, School of Health Sciences, Tokyo International University, Saitama, Japan; 3 Graduate School of Human Health Sciences, Tokyo Metropolitan University, Tokyo, Japan; 4 Department Physical Therapy, Hiroshima Cosmopolitan University, Hiroshima, Japan; 5 Department of Rehabilitation, Sendai Seiyo Gakuin University, Sendai, Japan

**Keywords:** Cognitive neuroscience, Learning and memory, Motor control

## Abstract

The visual-motor illusion (VMI) induces a kinesthetic illusion by watching one’s physically-moving video while the body is at rest. It remains unclear whether the early stages (immediately to one hour later) of motor learning are promoted by VMI. This study investigated whether VMI changes the early stages of motor learning in healthy individuals. Thirty-six participants were randomly assigned to two groups: the VMI or action observation condition. Each condition was performed with the left hand for 20 min. The VMI condition induced a kinesthetic illusion by watching one’s ball-rotation task video. The action observation condition involved watching the same video as the VMI condition but did not induce a kinesthetic illusion. The ball-rotation task and brain activity during the task were measured pre, post1 (immediately), and post2 (after 1 h) in both conditions, and brain activity was measured using functional near-infrared spectroscopy. The rate of the ball-rotation task improved significantly at post1 and post2 in the VMI condition than in the action observation condition. VMI condition lowers left dorsolateral prefrontal cortex and right premotor area activity from post1 to pre compared to the action observation condition. In conclusion, VMI effectively aids early stages of motor learning in healthy individuals.

## Introduction

The establishment of human motor learning or the acquisition of skills requires repetitive physical practice^1–3^, and various methods have been reported to promote motor learning (i.e., repetitive physical practice, action observation, and virtual reality)^3–5^. Motor learning is defined as relatively permanent changes in the capability of skilled behaviors resulting from practice or experience^6^. Motor learning can be promoted at an early or later stage, which distinguishes between fast (early stage of learning) and slow learning (learning processes that occur over longer time spans)^7^. Lohse et al.^8^ divided the motor learning period into short (< 1 h), medium (> 1 and < 24 h), and long (> 24 h to 5 weeks) periods and investigated differences in brain activity using meta-analysis. They reported that the prefrontal and premotor cortices (PMC) and inferior parietal lobules decreased at all time scales. Motor learning can be characterized as an improvement in motor performance accompanied by changes in neural activity.

Recently, it was reported that action observation facilitates the early stages of motor learning^9–11^ and changes brain activity in healthy individuals^12–14^. Action observation (AO) is a method in which motor function and learning are promoted by observing movements (i.e., hand movement and balance)^12–14^. The underlying mechanism involves the mirror neuron system^15^, which is activated by imitating the movements of another person or oneself and motor imagery, among other various methods^12, 16, 17^. Emuk et al.^12^ examined whether observing videos of upper limb movements changed upper limb motor performance in healthy individuals. They show that the upper limb performance immediately changed after observing the video compared to other conditions. Nagai and Tanaka^13^ examined participants by observing a video of hand movements and measuring the activity of the sensorimotor area using electroencephalography. They reported that the activity of the sensorimotor area was higher when participants observed a video of hand movements. Moreover, it has been reported that action observation with a kinesthetic illusion (i.e., visual-motor illusion) changes motor function and brain activity in the primary motor cortex (M1) in healthy individuals^18–20^. These previous studies have demonstrated that AO can promote motor learning by facilitating improvements in various motor performances associated with the activation of the mirror neuron system.

Visual-motor illusion (VMI) induces a kinesthetic illusion by watching a first-person video of one’s physical movement while the body is at rest^21^. VMI is a method of using kinesthetic illusion induced by visual stimuli, such as mirror therapy, without moving the opposite side as in mirror therapy^22^. VMI evokes a sense of body ownership and agency by watching a physically moving video of healthy individuals and patients with stroke^21, 23, 24^. Dilena et al.^25^ investigated whether VMI increased the excitability of M1 compared with action observation using a systematic review. In accordance with all three selected studies, VMI was found to increase the excitability of the M1 compared with AO. Sakai et al.^21^ reported whether VMI and AO changed resting-state functional connectivity using functional near-infrared spectroscopy (fNIRS) in healthy individuals. They showed that VMI immediately changes the frontoparietal network and motor execution network compared with AO. However, it remains unclear whether VMI promotes the early stages of motor learning. If neurophysiological changes (i.e., increased brain activity) are induced by VMI, as shown in previous studies^25^, then motor performance may also improve immediately and promote the early stage of motor learning. Therefore, this study aimed to investigate whether VMI immediately changes motor performance and the early stages of motor learning in healthy individuals. The participants were randomly assigned to the VMI or AO group and performed the task for 20 min. The measurements were recorded for Pre, Post1 (immediately), and Post2 (after 1 h) for each group, in addition to the assessment of the ball-rotation task and monitoring of brain activity during the task.

## Results

Thirty-six healthy individuals were enrolled in this study (mean age: 24.4 ± 3.7 years; 17 males and 19 females). Four participants in the VMI group who did not experience the kinesthetic illusion (seven-point Likert scale: less than + 1 point) and three participants in the AO group who experienced the kinesthetic illusion (seven-point Likert scale: more than + 1 point) were excluded, resulting in a total of 29 included participants for analysis (VMI group: 14 participants, AO group: 15 participants). The two groups had no significant differences in basic characteristics (p > 0.05, Table 1).

**Table 1:** Characteristics of overall participants and two group.

Variables	Overall (N = 36)	VMI group	AO group	p value
		(N = 14)	(N = 15)	
Height (cm)	164.0 ± 8.3	165.1 ± 7.7	163.3 ± 9.1	p = 0.813
Weight (Kg)	56.6 ± 9.7	57.8 ± 10.6	55.8 ± 10.7	p = 0.683
Age (years)	24.4 ± 3.7	24.6 ± 4.0	24.3 ± 3.7	p = 0.847
Sex (male/female)	17/19	9/5	6/9	p = 0.191
The Edinburgh Handedness (Right, %)	79.3 ± 12.4	79.3 ± 13.3	78.0 ± 11.6	p = 0.683
Hand length (cm)	17.5 ± 1.0	17.8 ± 0.9	17.3 ± 1.1	p = 0.184

Data are expressed as mean ± standard deviation.

VMI, visual-motor illusion; AO, action observation.

**Figure 1:**
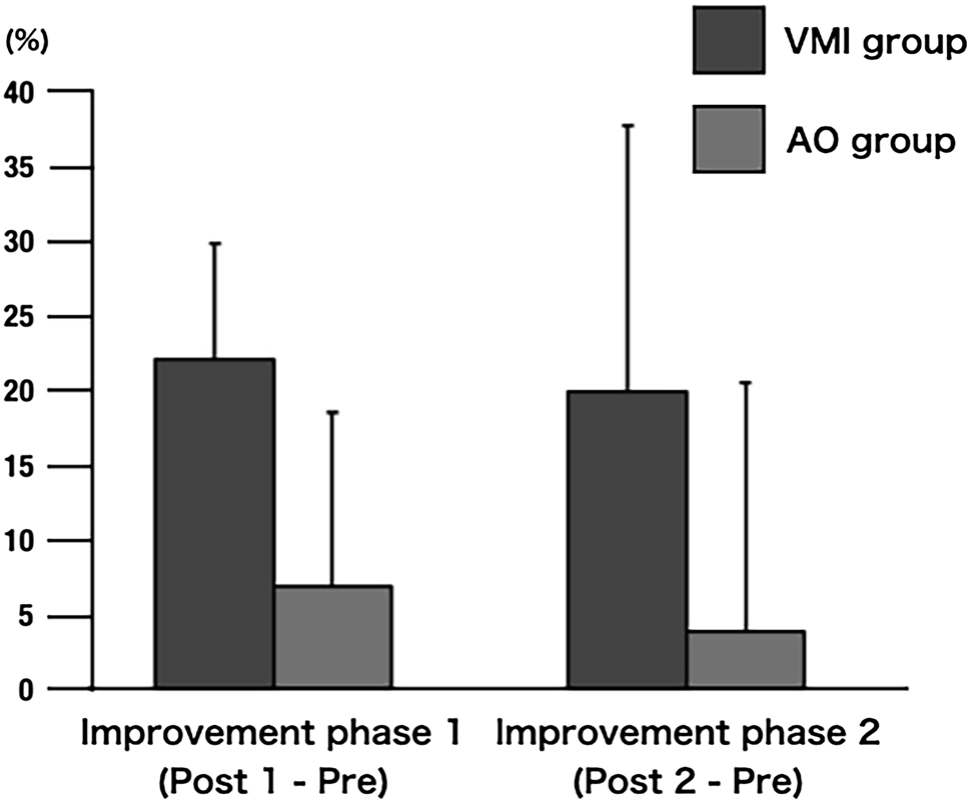
The ball rotation task. Dark gray indicated the visual-motor illusion (VMI) group. Light gray indicated the action observation (AO) group. The main effect (between the VMI and AO groups) was observed [F (1, 54) = 6.09, p < 0.001], and the VMI group showed a significantly higher finding than the AO group. The phase main effect (between the improvement phases 1 and 2) did not show a significant difference [F (1, 54) = 0.47, p = 0.495], and the interaction did not show a significant difference between the improvement phases 1 and 2 [F (1, 54) = 0.01, p = 0.911].

Regarding the improvement rate in the ball-rotation task, the group main effect (between the VMI and AO groups) was observed [F (1, 54) = 6.09, p < 0.001], and the VMI group showed significantly higher values than the AO group in both phases. The phase main effect (between improvement phases 1 and 2) was not observed [F (1, 54) = 0.47, p = 0.495], and no interaction was observed between the improvement phases and groups [F (1, 54) = 0.01, p = 0.911, Fig. 1].

**Figure 2:**
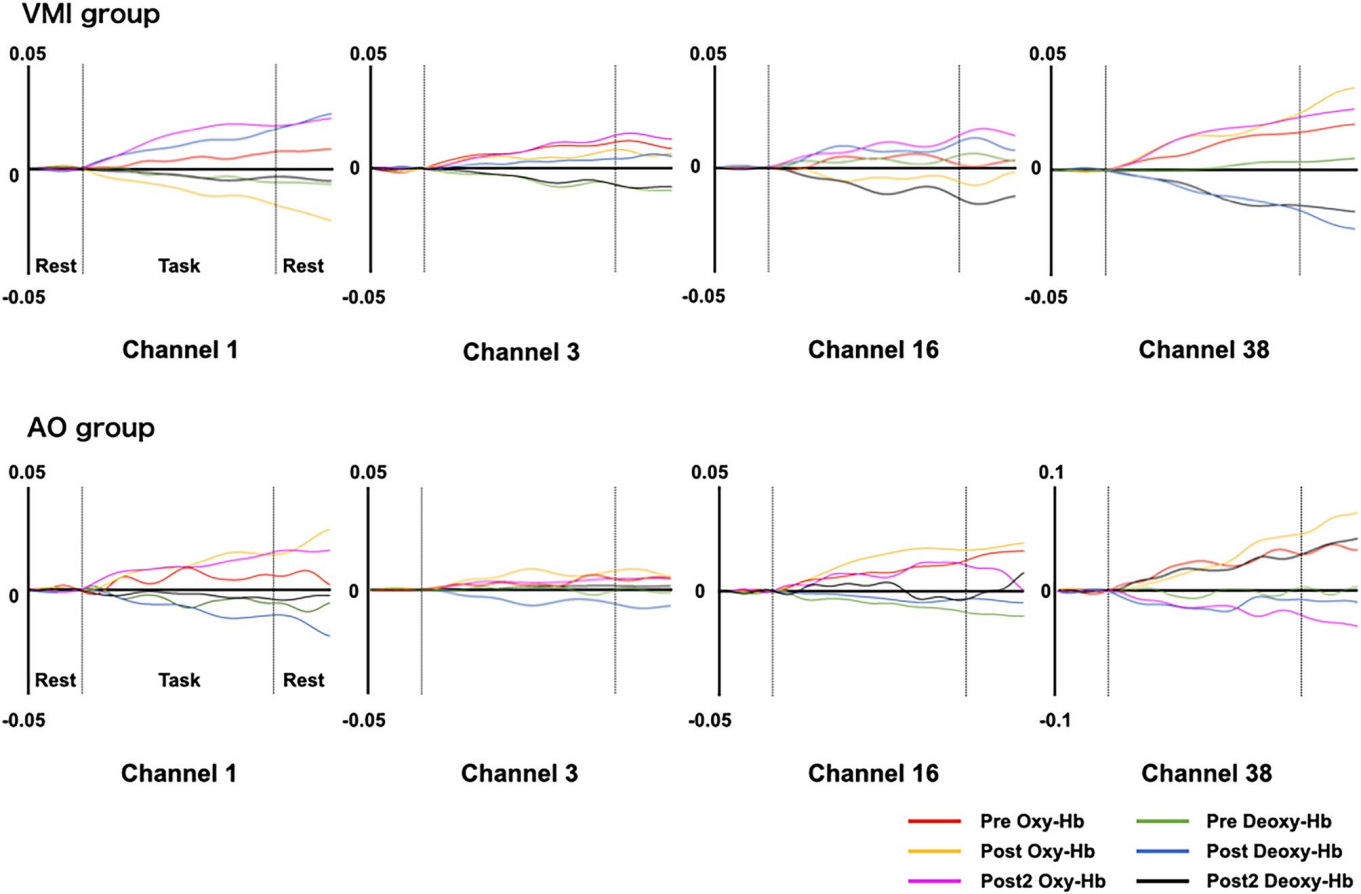
Signal responses of oxygenated hemoglobin and deoxygenated hemoglobin in the significant channels. Red line indicated oxygenated hemoglobin (oxy-Hb) in pre phase. Light green line indicated deoxygenated hemoglobin (deoxy-Hb) in pre phase. Yellow line indicated oxy-Hb in post phase. Blue line indicated deoxy-Hb in post phase. Pink line indicated oxy-Hb in post 2 phase. Black line indicated deoxy-Hb in post 2 phase.

Figure 2 shows the signal responses of oxy-Hb and deoxy-Hb in significant channels. Regarding brain activity, the left DLPFC (Ch1) and right PMC (Ch21) of the VMI group significantly decreased compared to those of the AO group in improvement phase 1 (p < 0.05, FDR-corrected, Table 2). Furthermore, the left DLPFC (Ch1) of the VMI group in improvement phase 2 was significantly higher than in improvement phase 1 (p < 0.05, FDR-corrected, Table 3). In contrast, the right DLPFC (Ch3) of the VMI group in improvement phase 2 was significantly higher than that of the AO group (p < 0.05, FDR-corrected, Table 2). In the AO group, the right PMC (Ch21) and left Pa (Ch38) in improvement phase 2 were significantly lower than those in improvement phase 1 (p < 0.05, FDR-corrected; Table 3).

**Table 2:** Results of brain activity of both group.

Phase	Channels	ROI	VMI group	AO group	p value
Improvement phase 1	Ch 1	Left DLPFC	− 0.025 ± 0.036	0.029 ± 0.054	p < 0.05
Improvement phase 1	Ch 21	Right PMC	− 0.014 ± 0.038	0.021 ± 0.035	p < 0.05
Improvement phase 2	Ch 3	Right DLPFC	0.001 ± 0.016	− 0.001 ± 0.015	p < 0.05

Data are expressed as mean ± standard deviation.

VMI, visual-motor illusion; AO, action observation; Ch, channels; ROI, region of interest; DLPFC, dorsolateral prefrontal cortex; PMC, premotor cortex; p values, false discovery rate corrected.

**Table 3:** Results of brain activity of improvement phase.

Group	Channels	ROI	Improvement phase 1	Improvement phase 2	p value
VMI group	Ch 1	Left DLPFC	− 0.025 ± 0.036	0.001 ± 0.019	p < 0.05
AO group	Ch 21	Right PMC	0.021 ± 0.035	− 0.006 ± 0.028	p < 0.05
AO group	Ch 38	Left Pa	0.051 ± 0.089	− 0.019 ± 0.082	p < 0.05

Data are expressed as mean ± standard deviation.

VMI, visual-motor illusion; AO, action observation; Ch, channels; ROI, region of interest; DLPFC, dorsolateral prefrontal cortex; PMC, premotor cortex; Pa, parietal area; p values, false discovery rate corrected.

Regarding the degree of kinesthetic illusion, Q1 was 1.75 (1  to  3) with the VMI group and − 0.5 (− 3  to  0) with the AO group. The VMI group had significantly higher scores than the AO group (Z = 8.23, p < 0.001). Regarding the degree of sense of body ownership, Q2 was 2 (1  to  3) with the VMI group and − 2 (− 3  to  1) with the AO group, and Q3 was − 2 (− 3  to  − 1) with the VMI group and 2 (− 1  to  3) with the AO group. The VMI group had significantly higher scores than the AO group (Q2: Z = − 4.40, p < 0.001; Q3: Z = 4.40, p < 0.001). Q4 was − 2 (− 3  to  − 1) with the VMI group and − 1 (− 3  to  1) with the AO group, and there were no significant differences between the two groups (Z = 1.37, p > 0.05). Regarding the degree of sense of agency, Q5 was 2 (1  to  3) with the VMI group and − 1 (− 3  to  3) with the AO group, and Q6 was − 1.5 (− 3  to  − 3) with the VMI group and 1 (− 3  to  3) with the AO group. The VMI group had significantly higher scores than the AO group (Q5: Z = − 3.22, p < 0.001; Q6: Z = 2.62, p = 0.009).

## Discussion

In the current study, we investigated whether VMI immediately changes motor performance and early stages of motor learning in healthy individuals. As a result, compared to pre, VMI promoted the early stage of motor learning with a higher improvement rate in ball-rotation task over post1 (immediately) and post2 (after 1 h) than the AO group. In addition, the VMI group in improvement phase 1 (post1−pre) showed significantly decreased brain activity in the left DLPFC and right PMC compared to the AO group. These results indicate that VMI immediately changes motor performance and brain activity and promotes the early stages of motor learning.

The VMI group of improvement phases 1 and 2 (post1−pre and post2−pre) had a significantly higher improvement rate in the ball rotation task than the AO group. This result supports the findings of a previous study^16^. Nojima et al.^16^ reported that various visual stimulations (i.e., AO with a third perspective, AO with a first perspective, and VMI) change motor performance and promote motor learning using the ball-rotation task. They reported that VMI immediately changed the number of ball rotations and promoted motor learning compared to other AOs. They also reported that the videos were the same for AO and VMI; the difference was whether a kinesthetic illusion was induced. Similar to previous study, the current study used the same videos for the VMI and AO groups, and the difference between the two conditions was whether they were illusory. Therefore, the kinesthetic illusions may have caused an immediate change in the motor performance. In addition, we used a seven-point Likert scale to measure the degree of kinesthetic illusion to clarify whether this was an effect of kinesthetic illusion. The results showed that the VMI group had a significantly higher degree of kinesthetic illusion than the AO group. Therefore, we believe that the high improvement rate of ball rotation was due to the kinesthetic illusion. Moreover, the effect of the ball rotation task in the VMI group may be associated with a sense of body ownership and agency. VMIs have been reported to induce a sense of body ownership and agency^21, 24, 35^. VMI placed the participant’s actual hand under the monitor, and the hand on the monitor was the same size as the actual hand. This facilitates the matching of visual and somatosensory sensations and a sense of body ownership^36, 37^. A sense of body ownership is induced if the body is spatially congruent and the actual hand is near the video^21, 23, 36, 37^. Regarding the sense of agency, it has been reported that the sense of agency is induced by the congruence between the prediction by motor intention (efferent copy) and the actual sensory feedback information^38, 39^. However, some reports indicate that a sense of agency is facilitated when a sense of body ownership is elicited even when motor intention is low^40, 41^, and the mechanism is unclear. VMI induces a kinesthetic illusion that evokes a sensation of movement as if the participant is not moving. Thus, the participants may have unconsciously had a motor intention and felt a sense of agency because of the induced kinesthetic illusion and sense of body ownership^41^. Further research is needed on the relationship between VMI and sense of agency.

Regarding brain activity, the left DLPFC (Ch1) and right PMC (Ch21) of the VMI group were significantly decreased compared to those of the AO group during improvement phase 1. This result supports the findings of previous studies^8, 42^. It has been reported that the brain activity of the DLPFC and PMC decreased with motor learning^8, 42^. The DLPFC is involved in top-down attention, working memory, and executive functions such as planning and monitoring^43^. In addition, the DLPFC recognizes the most critical neural bases underlying cognitive processing during the early stages of motor learning^44, 45^. In particular, left DLPFC was associated with attention control^46^. In addition, the PMC is involved in the temporal organization of sequential movements, motor selection, and generation of motor sequences that conform to a correct plan from memory, and its activity decreases with motor learning^42, 47^. The VMI group of improvement phases 1 and 2 had a significantly higher improvement rate in the ball-rotation task than the AO group. Therefore, facilitated motor learning reduced brain activity in the left DLPFC and right PMC because ball rotation became automatic and no longer required attentional control. In contrast, the left DLPFC (Ch1) of the VMI group in improvement phase 2 was significantly higher than that in improvement phase 1. This finding is not consistent with that from a previous study^8^. This might have been related to a lack of practice, motor learning speed, or established maintenance of skills. It was reported that the DLPFC increased when the task was under-practiced or motor skills were retained^48, 49^. Our protocol was a 20-min kinesthetic illusion followed by a 1-h rest. Therefore, one potential reason for the reported finding is that the lack of practice due to the lack of time in the kinesthetic illusion condition and the effect of the break led to higher DLPFC activity to perform the skill.

In the AO group, the right PMC (Ch21) and left Pa (Ch38) in improvement phase 2 were significantly lower than those in improvement phase 1. A previous study reported that the right DLPFC and left Pa decreased with motor learning^8^. However, motor learning speed depends on the task type and difficulty^50^. In the early motor learning, body representations have the advantage of motor execution compared with observational learning^50^. It was reported that VMI immediately activate the motor execution network^21^. Therefore, the VMI group showed faster learning than the observation group, which showed a slightly slower learning, speculating that brain activity was reduced in improvement phase 2 (post2−pre). The right DLPFC (Ch3) of the VMI group in improvement phase 2 was significantly higher than that in the AO group. This could be attributed to the significant difference caused by the motor learning speed in the two groups.

One limitation of this study is that it did not measure mid- to long-term motor performance or brain activity changes during VMI. Second, we did not measure actual motor performance conditions. Future research should include motor performance conditions and investigate the mid- to long-term changes in motor learning. Third, because fNIRS was used, it only measured brain activity in the surface layer of the cerebral cortex. The cerebellum is particularly involved in motor learning^8^. Therefore, it is assumed that the cerebellum is affected by VMI. In the future, we will measure changes in motor learning due to VMI using fMRI and other methods. Fourth, the sample size could have been small considering the characteristics of fNIRS. Therefore, further studies should be performed using larger sample sizes. Finally, the relationship between VMI and sense of agency is unclear. Therefore, these factors should be investigated further.

In conclusion, VMI changes motor function and brain activity and promotes the early stages of motor learning in healthy individuals.

## Methods

### Participants

Thirty-six healthy individuals participated in this study (mean age: 24.4 ± 3.7 years, 17 males and 19 females). All participants were right-handed, according to the Edinburgh Handedness Inventory^26^. All participants had no history of orthopedic or neurological diseases. The purpose of the study was explained to the participants, and written informed consent was obtained. This study was conducted with the approval of the Institutional Ethics Committee of Tokyo Metropolitan University (approval number: 21033) and complied with the ethical standards of the 1964 Declaration of Helsinki.

### Materials and methods

**Figure 3:**
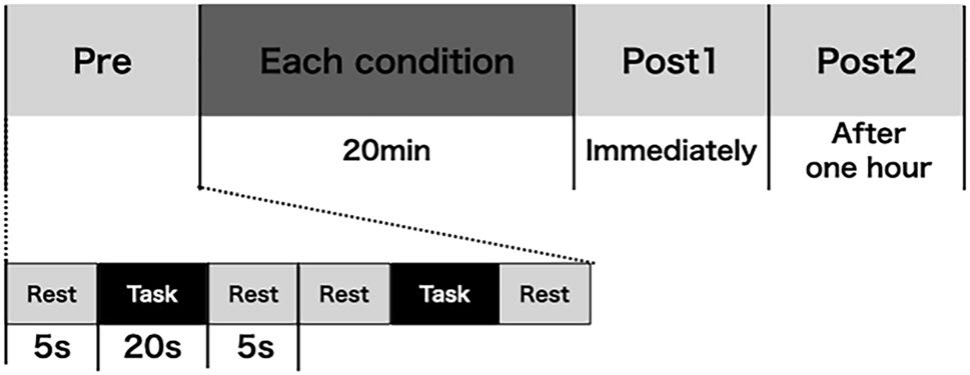
Protocol and measurement phase. Each condition was conducted for 20 min. Pre, Post1 (immediately), and Post2 (1 h later) each condition, the ball rotation task was measured using the block design, and the brain activity was measured during the ball rotation task using the functional near-infrared spectroscopy. The degree of kinesthetic illusion, sense of body ownership, and sense of agency were measured immediately after each condition.

Participants randomly performed the kinesthetic illusion condition with VMI (VMI group) or the action observation condition (AO group, 18 participants each). Each condition was performed for 20 min on the left upper limb. Pre, Post1 (immediately), and Post2 (after one hour) for each condition, the ball-rotation task was performed, and brain activity was measured during the ball-rotation task using fNIRS (Fig. 3). Immediately after each condition, the degree of kinesthetic illusion, sense of body ownership, and sense of agency were used to measure the extent to which each condition evoked the kinesthetic illusion, sense of body ownership, and sense of agency, respectively.

### Conditions

In each group, the participants were seated in a chair with their left upper limb placed on a table, which was maintained in the resting position.

**Figure 4:**
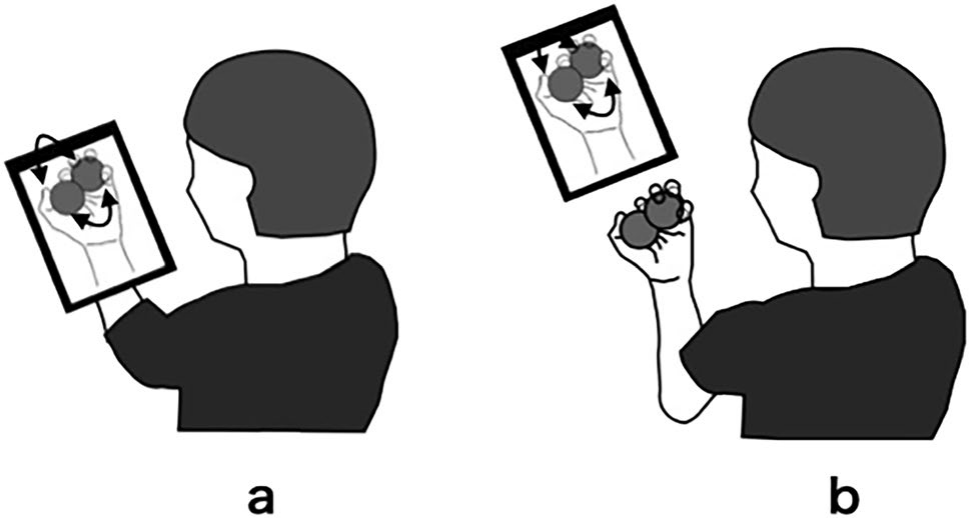
Set up of each condition. Participants were seated in a chair with their left upper limb placed on a table and maintained in a resting position. (**a**) The Visual-motor illusion group observed a video of their counterclockwise ball rotation task. (**b**) The action observation group watched the same video as the visual-motor illusion group. However, the participants’ left hand was positioned in front of the video, which made it more difficult to induce a kinesthetic illusion while the video was observed.

The kinesthetic illusion condition with the VMI consisted of a video of the participant’s left hand performing a clockwise ball-rotation task (Fig. 4a). In this video, a 38-mm-diameter ball was used, and the participants were asked to practice rotating the ball clockwise as fast as possible for 10 min. It was then filmed using a tablet (iPad Pro, Apple, Cupertino, CA, USA) and edited to 1.5 × using an application^10^. It has been reported that a 1.5 × video is most likely to facilitate the early stages of motor learning^10^. For the match between the viewed and real hands, the participant superimposed the real hand on top of the viewed hand to make them the same size. Subsequently, the participant’s left hand was positioned to overlap the video of the left hand, as shown in the video. During the AO condition, the same video presented as in the kinesthetic illusion condition with VMI was used (Fig. 4b). However, the AO condition was performed with the participant’s left upper limb positioned in front of the video, such that the kinesthetic illusion was not easily induced and the video was observed^21, 23^.

### Assessments

The assessments included a ball-rotation task, brain activity during the ball-rotation task, kinesthetic illusion, sense of body ownership, and sense of agency using a seven-point Likert scale.

The ball-rotation task was performed with a 38-mm-diameter ball, and the participants were asked to perform it as quickly as possible with their left hand without letting the ball fall. The participants sat in a chair and performed the task on a table. A block design was used to simultaneously measure the brain activity in two sets: rest (5 s), task (20 s), rest (5 s) (Fig. 3). The number of ball rotations was measured once the ball on the thumb side returned to its starting position. The number of ball rotations was calculated using a camera (120 Hz) placed over the left upper limb, and images were captured.

**Figure 5:**
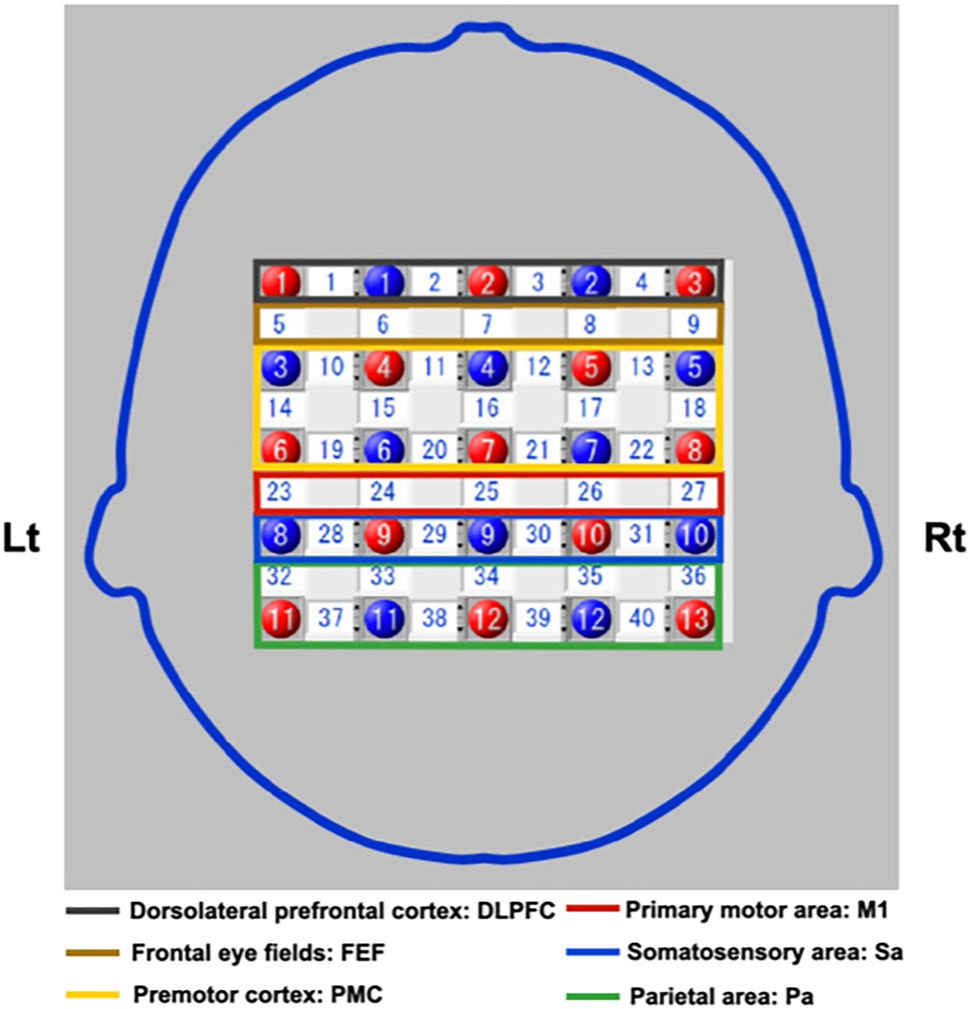
Region of interest using the functional near-infrared spectroscopy. The probe position was set to 40 channels using 25 probes (13 sources and 12 detectors) of 5 × 5. The probe distance was set at 3 cm. The regions of interest were the dorsolateral prefrontal cortex (DLPFC, channels 1–4), frontal eye field (FEF, channels 5–9), premotor cortex (PMC, channels 10–22), primary motor area (M1, channels 23–27), somatosensory area (Sa, channels 28–31), and parietal area (Pa, channels 32–40).

Brain activity was measured using fNIRS (LABNIRS, Shimadzu Co., Ltd., Kyoto, Japan). This device produced a continuous wave with wavelengths of 780, 805, and 850 nm. fNIRS measures oxygenated hemoglobin (oxy-Hb) and deoxygenated hemoglobin (deoxy-Hb) based on the modified Beer–Lambert law^27^. The oxy-Hb signal was used in many studies because it is more sensitive than the deoxy-Hb signal^28^. The probe position was set to 40 channels in total using 25 probes (13 sources and 12 detectors) of 5 × 5 (Fig. 5). The regions of interest (ROI) were the left and right dorsolateral prefrontal cortex (DLPFC), PMC, M1, primary somatosensory area (Sa), and parietal area (Pa). The probe distance was 30 mm, and the sampling rate was 10 Hz. For the full-head holder, the positions of the probes were determined to cover the top from the front using a 10–20 system centered on the central zone.

The seven-point Likert scale was used to assess the degree of kinesthetic illusion, sense of body ownership, and sense of agency^21, 23, 29^. The kinesthetic illusion was assessed using the phrase (Q1) “I feel my hand is moving,” and the responses on the seven-point Likert scale were as follows: “'I do not agree” (− 3), “I cannot say” (0), and “I agree” (+ 3). The degree of the sense of body ownership in the moving video image was assessed using the following three statements: (Q2) “I feel it is my own,” (Q3) “I feel it is not mine,” and (Q4) “I feel it is somebody else’s,” and the responses on the seven-point Likert scale were as follows: “I do not agree” (− 3), “I cannot say” (0), and “I agree” (+ 3). The degree of the sense of agency in the moving video image was evaluated using the following two statements: (Q5) “I feel it like I am in control of it” and (Q6) “I feel it is out of my control,” and the responses on the seven-point Likert scale were as follows: “I do not agree” (− 3), “I cannot say” (0), and “I agree” (+ 3).

### Analysis

To observe the effects of the early stages of motor learning, we analyzed the improvement rate and changes in the ball-rotation task and brain activity using improvement phases 1 (pre to post1) and 2 (pre to post2).

The number of ball rotations was calculated as the average of the two rotations, and the improvement rate was calculated using the following formula: Formula: (post1 or post2−pre)/pre × 100 (pre to post1: improvement phase 1; pre to post2; improvement phase 2).

The seven-point Likert scale was used to calculate the median, maximum, and minimum values. Regarding brain activity, the oxy-Hb data that contained a low signal-to-noise ratio in the source and detector placement were removed^30^. The oxy-Hb value of the rest and task phases was calculated by applying an oxy-Hb value to the 0.01–0.1 Hz bandpass filter^31^, calculating the task minus rest value, and calculating the average value of the two sets. The pre-value was then subtracted from post1 or post2 to observe the changes from pre (pre to post1: improvement phase 1; pre to post2: improvement phase 2). For the identification of ROIs, all the channels were referenced to international 10–20 system landmarks (nasion, inion, right, and left preauricular points) and were recorded with a three-dimensional (3D) digitizer (3 SPACE®, FASTRAK®, Polhemus Co., Ltd., Colchester, VT, USA) to determine which brain regions corresponded to the positions of each channel. All channels then converted these coordinates into the locations of 40 channels based on the estimated Montreal Neurological Institute (MNI) space using NIRS-SPM^32, 33^. NIRS-SPM transforms the functional image to MNI space using probabilistic registration in reference to 3D digitized data of all channels and landmark positions with the international 10–20 system^31, 33^. The results demonstrated that the ROIs included the left DLPFC (channels 1, 2), right DLPFC (channels 3, 4), left PMC (channels 10, 11, 14, 15, 19, 20), right PMC (channels 12, 13, 17, 18, 21, 22), left M1 (channels 23, 24), right M1 (channels 26, 27), left Sa (channels 28, 29), right Sa (channels 30, 31), left Pa (channels 32, 33, 37, 38), and right Pa (channels 35, 36, 39, 40).

### Statistical analyses

Statistical analyses were performed using the SPSS software version 28 (IBM Corp., Armonk, NY, USA).

Regarding the improvement rate in the ball-rotation task, we conducted a two-factor (group and phase) analysis of variance (p < 0.05). For the seven-point Likert scale, we conducted the Mann–Whitney U test. For brain activity, in accordance with normality, the paired t-test, non-paired t-test, Mann–Whitney U test, or Wilcoxon signed-rank test were used to compare the differences between the groups (the VMI and AO groups), phase (improvement phases 1 and 2), and ROIs. Multiple tests were controlled using the false discovery rate (FDR) (p < 0.05)^34^.

## Data Availability

The datasets generated during and/or analyzed during the current study are available from the corresponding author on reasonable request.
